# Genetic Analysis of DinG Family Helicase YoaA and Its Interaction with Replication Clamp Loader Protein HolC in Escherichia coli

**DOI:** 10.1128/JB.00228-21

**Published:** 2021-08-20

**Authors:** Vincent A. Sutera, Thalia H. Sass, Scott E. Leonard, Lingling Wu, David J. Glass, Gabriela G. Giordano, Yonatan Zur, Susan T. Lovett

**Affiliations:** a Department of Biology, Brandeis University, Waltham, Massachusetts, USA; b Rosenstiel Basic Medical Sciences Research Center, Brandeis University, Waltham, Massachusetts, USA; Princeton University

**Keywords:** DNA damage response, DNA polymerase, DNA repair

## Abstract

The XP-D/DinG family of DNA helicases contributes to genomic stability in all three domains of life. Here, we investigate the role of one of these proteins, YoaA, of Escherichia coli. In E. coli, YoaA aids in tolerance to the nucleoside azidothymidine (AZT), a DNA replication inhibitor, and physically interacts with a subunit of the DNA polymerase III holoenzyme, HolC. We map the residues of YoaA required for HolC interaction to its C terminus by yeast two-hybrid analysis. We propose that this interaction competes with HolC’s interaction with HolD and the rest of the replisome; YoaA indeed inhibits growth when overexpressed, dependent on this interaction region. By gene fusions, we show that YoaA is repressed by LexA and induced in response to DNA damage as part of the SOS response. Induction of YoaA by AZT is biphasic, with an immediate response after treatment and a slower response that peaks in the late log phase of growth. This growth-phase-dependent induction by AZT is not blocked by *lexA3* (Ind^−^), which normally negates its self-cleavage, implying another means to induce the DNA damage response that responds to the nutritional state of the cell. We propose that YoaA helicase activity increases access to the 3′ nascent strand during replication; consistent with this, YoaA appears to aid in the removal of potential A-to-T transversion mutations in *ndk* mutants, which are prone to nucleotide misincorporation. We provide evidence that YoaA and its paralog DinG may also initiate template switching that leads to deletions between tandem repeats in DNA.

**IMPORTANCE** Maintaining genomic stability is crucial for all living organisms. Replication of DNA frequently encounters barriers that must be removed to complete genome duplication. Balancing DNA synthesis with its repair is critical and not entirely understood at a mechanistic level. The YoaA protein, studied here, is required for certain types of DNA repair and interacts in an alternative manner with proteins that catalyze DNA replication. YoaA is part of the well-studied LexA-regulated response to DNA damage, the SOS response. We describe an unusual feature of its regulation that promotes induction after DNA damage as the culture begins to experience starvation. Replication fork repair integrates both DNA damage and nutritional signals. We also show that YoaA affects genomic stability.

## INTRODUCTION

The YoaA protein of Escherichia coli is a member of the XP-D/DinG family of DNA helicases, with members found in all three domains of life. These are superfamily 2 helicases with the shared property of 5′-to-3′ translocation on single-strand DNA (ssDNA) and an intrinsic Fe-S cluster. In humans, these proteins play various roles in DNA repair and the maintenance of genomic stability, the loss of which results in a variety of genetic diseases (1–4).

The bacterium Escherichia coli encodes two paralog proteins of this family, DinG and YoaA, both of which appear to be induced by DNA damage as part of the SOS response (5–7). DinG encodes a structure-specific DNA helicase with the ability to unwind D-loops, R-loops, and G-quadruplex sequences (8–10). Despite its induction by UV irradiation, *dinG* mutants show only slight sensitivity to UV (8). Along with two SF1 helicase proteins, UvrD and Rep, DinG appears to enhance the survival of head-on replication/transcriptional collisions *in vivo* (11), when highly transcribed regions of the chromosome are inverted.

YoaA was identified in a genetic screen for factors that promote tolerance to the chain-terminating nucleoside azidothymidine (AZT) in Escherichia coli (12). AZT is incorporated during DNA replication and, since it blocks DNA chain elongation, produces ssDNA gaps at the replication fork; cells can tolerate certain levels of AZT through its removal from DNA by exonuclease III (13) or DnaQ proofreading (12). Mutants of *yoaA* are viable but are strongly sensitive to AZT (12) as well as to methyl methanesulfonate (MMS) (14). Mutants of *dinG* are only very slightly AZT sensitive but further enhance the sensitivity of *yoaA* mutants when combined.

YoaA is of particular interest because it physically interacts with the replisome protein HolC (χ) of DNA polymerase III (Pol III) (12, 15, 16). Increased expression of HolC, like YoaA, promotes tolerance to AZT *in vivo* (12). HolC is purified as an intrinsic component of the DNA polymerase III, where it serves as an accessory protein to the clamp loader complex (17, 18). It is the one component of the replisome that interacts with single-strand DNA binding protein (SSB) (19). In addition to its interaction with SSB, HolC forms a heterodimeric complex with HolD (ψ); it is HolD that links this accessory dimer to the clamp loader and to the rest of the replisome (20, 21).

The HolC/YoaA complex and the HolC/HolD complex appear to be mutually exclusive structures (16). The same residues buried at the HolC/HolD interface, F64 and W57 (22), are also required to form a complex with YoaA and essential for AZT tolerance *in vivo* (16). The expression of YoaA/HolC/HolD yields two complexes, HolC/HolD and HolC/YoaA, with no evidence of a ternary complex (16). This finding led to the hypothesis that HolC forms two complexes, HolC/HolD, dedicated to replication, and HolC/YoaA, dedicated to repair, both recruited to ssDNA through HolC’s SSB interaction.

In this study, we investigate further the genetic role of YoaA. Using yeast two-hybrid analysis, we map residues required for HolC interaction to the C-terminal 18 amino acids of YoaA and show that they are required for YoaA function *in vivo*. When it is strongly overexpressed and its C terminus is intact, YoaA inhibits growth. By gene fusions, we confirm LexA repression of YoaA expression and its induction by AZT; mutation of the putative LexA box 24 nucleotides upstream of the open reading frame yields constitutively high expression levels even in the absence of damage. A noninducible allele of LexA, *lexA3*, blocks the bulk of AZT induction of *yoaA* expression, although there remains some residual induction of P*yoaA* by AZT, especially during the transition of the culture from exponential growth to stationary phase, suggesting an alternative mechanism for overcoming LexA repression at the locus, induced by starvation. Consistent with our hypothesis that YoaA provides access to the 3′ nascent strand during replication, elevated YoaA expression promotes template switching that produces deletions between DNA tandem repeats and reduces T-to-A transversion mutations in an *ndk* mutant prone to nucleotide misincorporation.

## RESULTS

### Determination of HolC binding residues within YoaA.

**FIG 1:**
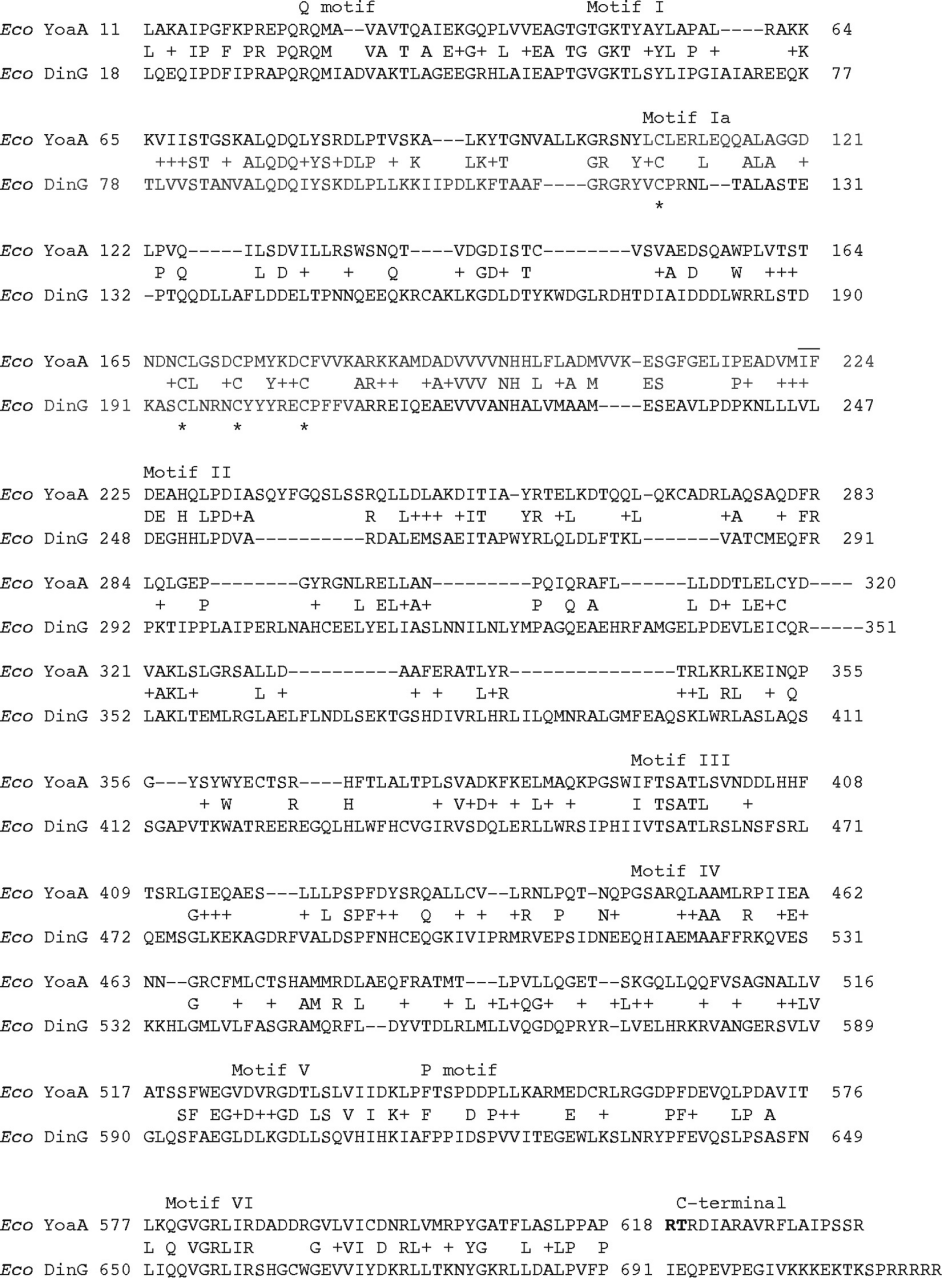
BLAST alignment of YoaA and DinG proteins. Conserved helicase motifs are indicated at the top, and cysteine residues of the FeS cluster are marked at the bottom with an asterisk. YoaA R619 and T620, implicated in HolC binding, are shown in boldface type at the C terminus.

The alignment of Escherichia coli DinG and YoaA proteins (Fig. 1) shows 29% identity over the length of the 2 proteins, including all helicase motifs (Q motif; motifs I, Ia, II, III, IV, V, and VI; and P motif), the 2 helicase HD1 and HD2 domains, and the 4 cysteine residues that coordinate Fe-S binding (23). In our previous study, we showed that K51 (Walker A, motif I), C168 (Fe-S cluster), and D225 (Walker B, motif II) (Fig. 1) were required for *yoaA* to promote AZT tolerance *in vivo* (12). The most diverged regions of the YoaA and DinG proteins are the arch domain (between motifs II and III) and the C terminus. A clue to the YoaA region that binds HolC came from previous pulldown experiments where we noted that a C-terminally truncated proteolytic fragment of YoaA present in the extracts failed to be pulled down with HolC as did full-length YoaA (12).

**FIG 2:**
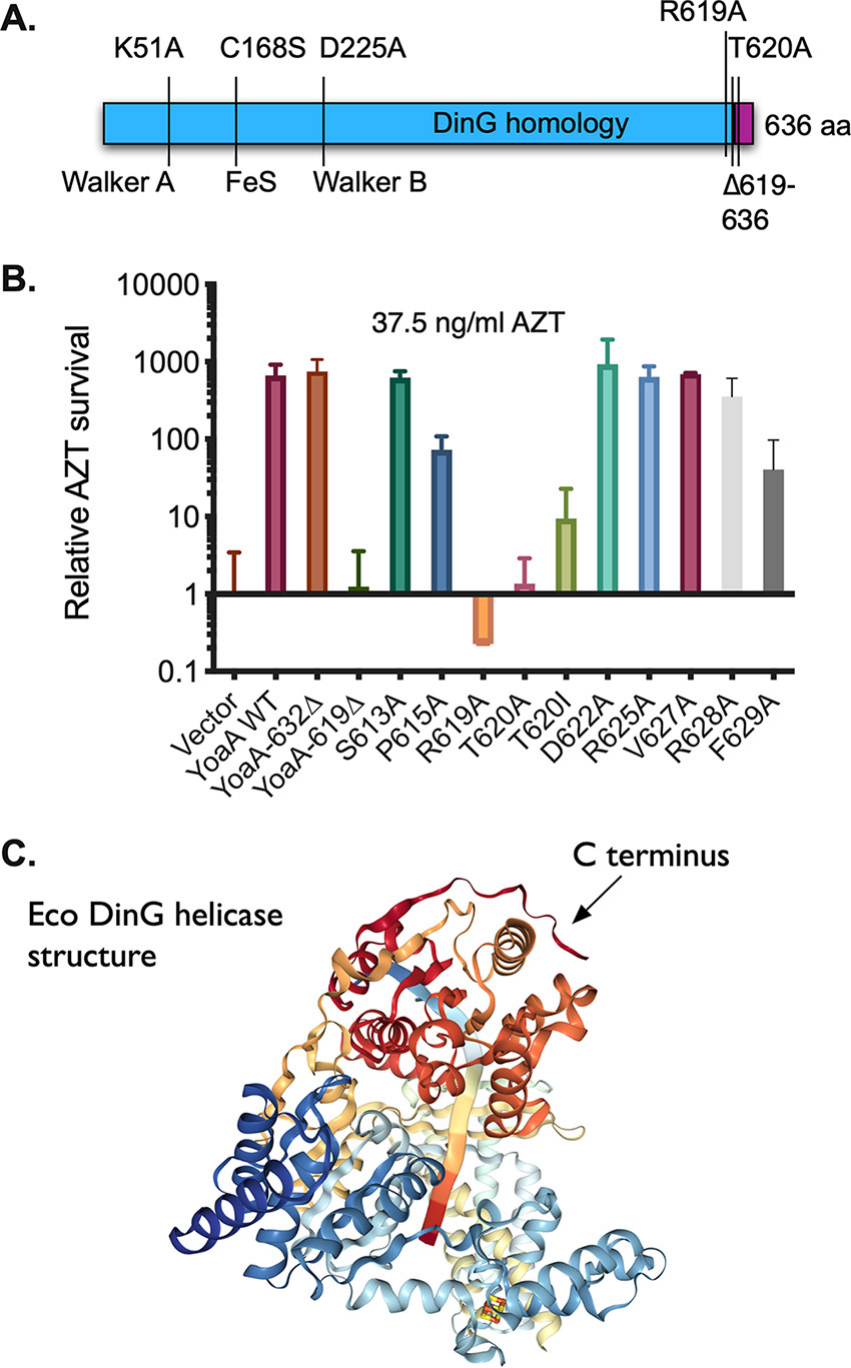
YoaA complementation assays. (A) Schematic of the *yoaA* gene showing previously identified noncomplementing mutations (12) and those identified in this study. The C-terminal region dissimilar from that of E. coli (Eco) DinG is indicated in purple. aa, amino acids. (B) Complementation assay. The plating efficiency at 37.5 ng/ml AZT of strains carrying the indicated *yoaA* plasmid alleles in a *yoaA*Δ strain was determined relative to the vector control. Fractional survival values at this dose were 0.66 for *yoaA*^+^ and 0.0009 for the vector control. Error bars represent standard deviations. (C) E. coli DinG structure. The image is from RCSB PDB accession number 6FWR (23), with the C terminus (darkest red) indicated with an arrow. DNA within the structure is shown as a flat ribbon.

We deleted the C-terminal amino acids of YoaA on a plasmid-expressed His_6_-tagged allele and assayed its ability to complement the AZT sensitivity phenotype of *yoaA* mutants (Fig. 2). (Although the *yoaA* gene is transcribed from the *tac* promoter on these plasmids, we were able to detect complementation without the addition of the inducer isopropyl-β-d-thiogalactopyranoside [IPTG].) Whereas the wild-type (wt) allele and *yoaA*Δ632–636 enhanced survival at a 37.5-ng/ml dose of AZT, almost 1,000-fold relative to the plasmid vector control, the *yoaA*Δ619–636 allele failed to complement, with a plating efficiency on AZT medium similar to that of the vector control. We mutated a number of individual residues within the region between residues 619 and 631 and found that both R619A and T620A destroyed YoaA function as measured by AZT tolerance, whereas D622A, R625A, V627A, and R628A had no effect; F629A and T620I showed a partial loss of complementation.

**FIG 3:**
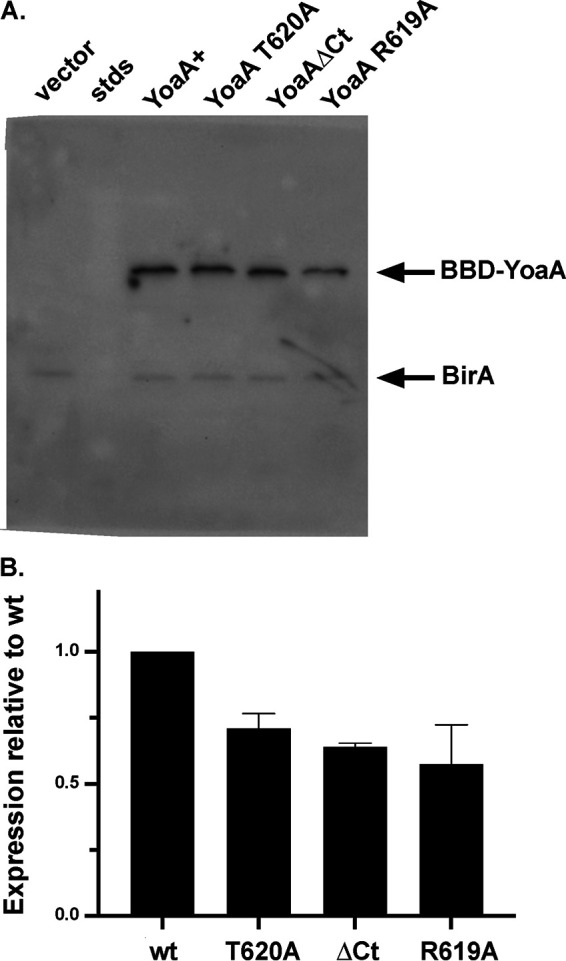
YoaA mutant protein expression. (A) Western blot of BBD-fused YoaA carrying the indicated alleles (“ΔCt” [C-terminal deletion] is Δ619–636 of YoaA) using neutravidin detection, compared to cells expressing the vector. BirA is the biotin binding protein of E. coli. A representative gel is shown. (B) Quantitation of Western blot data, showing averages and ranges from 2 independent experiments.

After inducing plasmid expression with IPTG, Western blotting of biotin binding domain-tagged YoaA, YoaAΔ619–636, YoaA R619A, and YoaA T620A showed levels of soluble YoaA protein comparable to those of the wild type, indicating that the failure to complement was not a result of protein degradation (Fig. 3). We confirmed that YoaAΔ619–636, YoaA R619A, and YoaA T620A conferred AZT sensitivity when transferred to the native, naturally expressed *yoaA* locus on the E. coli chromosome, showing that the dysfunction of these alleles is not plasmid dependent (see Fig. S1 in the supplemental material).

Based on its similarity with DinG, for which there is structural information, the location of the C terminus of YoaA is likely to be on the outside of the protein, at a site distinct from those involved in ATP and DNA binding. The corresponding C terminus of DinG is partially visible in crystal structures (PDB accession numbers 6FWS and 6FWR) on the exterior surface of the HD2 domain where it overlies helix 18, containing helicase motif IV (Fig. 2C [image from RCSB PDB accession number 6FWR {23}]).

**FIG 4:**
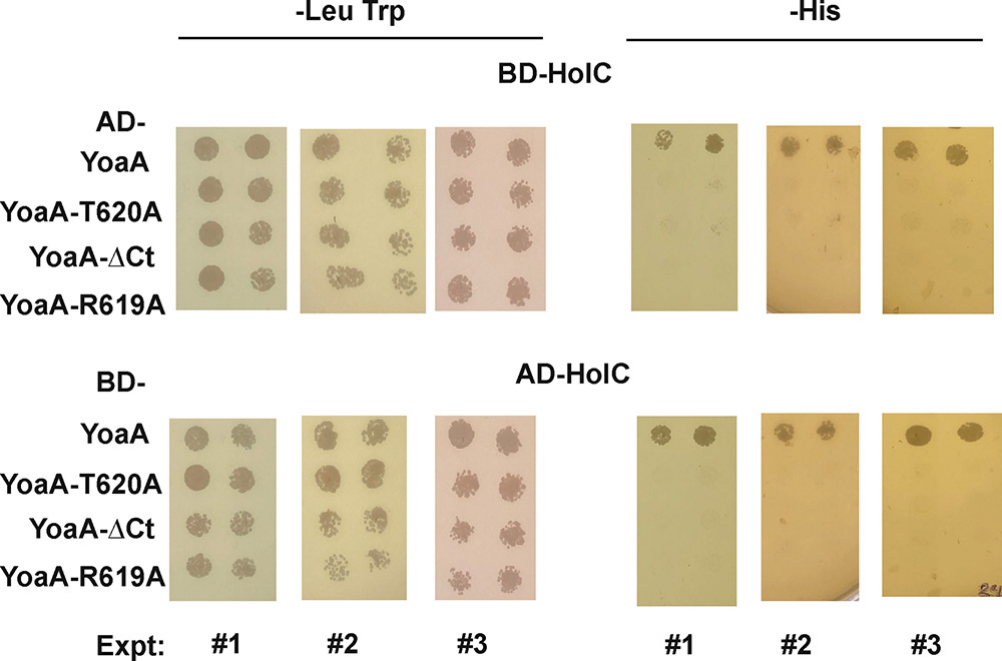
Yeast two-hybrid analysis of YoaA and HolC interactions. (Left) Segments of −Leu Trp plates that select for the two hybrid plasmids; (right) segments from −His plates that select for a functional interaction. The top row shows results from hybrids in which HolC is fused to the Gal4 DNA binding domain (BD) and YoaA (wt and three mutant alleles) is fused to the Gal4 activation domain (AD). YoaAΔCt is the YoaA truncation at amino acid 618, YoaAΔ619–636. In the bottom series, HolC is fused to the activation domain, and YoaA and its alleles are fused to the DNA binding domain. The results of three independent experiments are shown.

We transferred the most defective *yoaA* alleles to yeast two-hybrid fusions to ascertain whether these YoaA proteins retain the ability to interact with HolC, as we have demonstrated previously (12, 16) (Fig. 4). Whereas wt YoaA showed an interaction with HolC, indicated by growth on plates lacking His (−His plates), YoaA R619A, YoaA T620A, and the YoaAΔ619–636 C-terminal truncation did not. All strains grew equally well on plates lacking Trp and containing Leu (−Trp Leu plates), which select for the presence of the two plasmids. Control platings of individual plasmids combined with a vector control partner were performed in parallel and yielded negative results (data not shown), so the interaction requires both HolC and YoaA fusion partners. We obtained similar results regardless of whether the activation domain was fused to either HolC or YoaA, with the corresponding partner fused to the DNA binding domain, in three independent plating experiments (Fig. 4).

### Excess YoaA is toxic, dependent on an intact C terminus and Walker A box.

**FIG 5:**
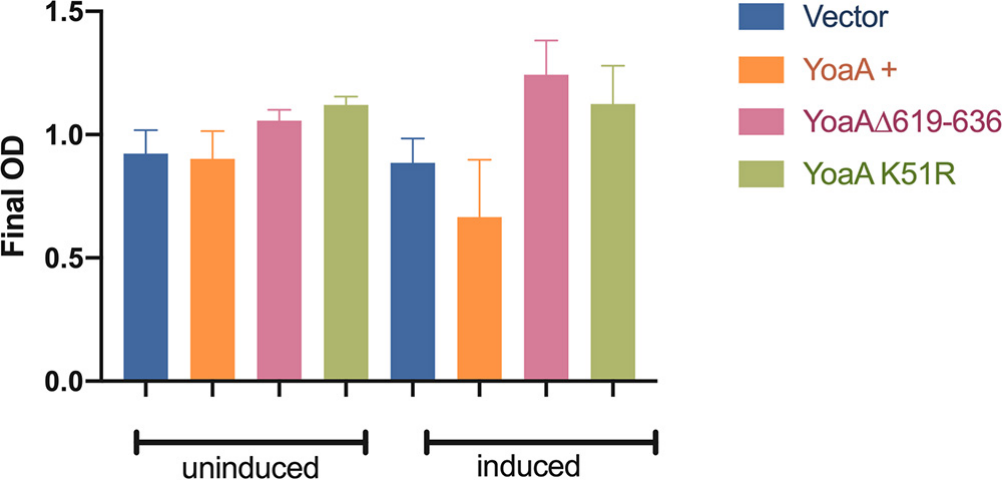
Growth inhibition by YoaA. YoaA was expressed from pCA24N-YoaA plasmids, with cultures split and then grown in LB (left) or LB with IPTG (right). Plotted are the final mean ODs of independent cultures (*n* = 18 for all but YoaA K51R, where *n* = 6) 3 h after the addition of IPTG, with error bars indicating standard deviations.

HolC binds to HolD, a second protein in the clamp loader complex of DNA Pol III, and both proteins are required to sustain full viability and fast growth (24). Because YoaA also forms a complex with HolC, as an alternative to that of HolC/HolD (16), excess YoaA might be expected to interfere with growth by competing with HolD for HolC. Induced, the expression of YoaA from a high-copy-number plasmid driven by the *tac* promoter was indeed found to inhibit the growth of otherwise wild-type strains (Fig. 5) (*P* = 0.005 by a *t* test), whereas uninduced cultures were not significantly unperturbed. A YoaA derivative lacking the C-terminal 18 amino acids required for interaction with HolC was not significantly toxic, nor was the K51R mutant of the Walker A box.

### Expression of YoaA.

**FIG 6:**
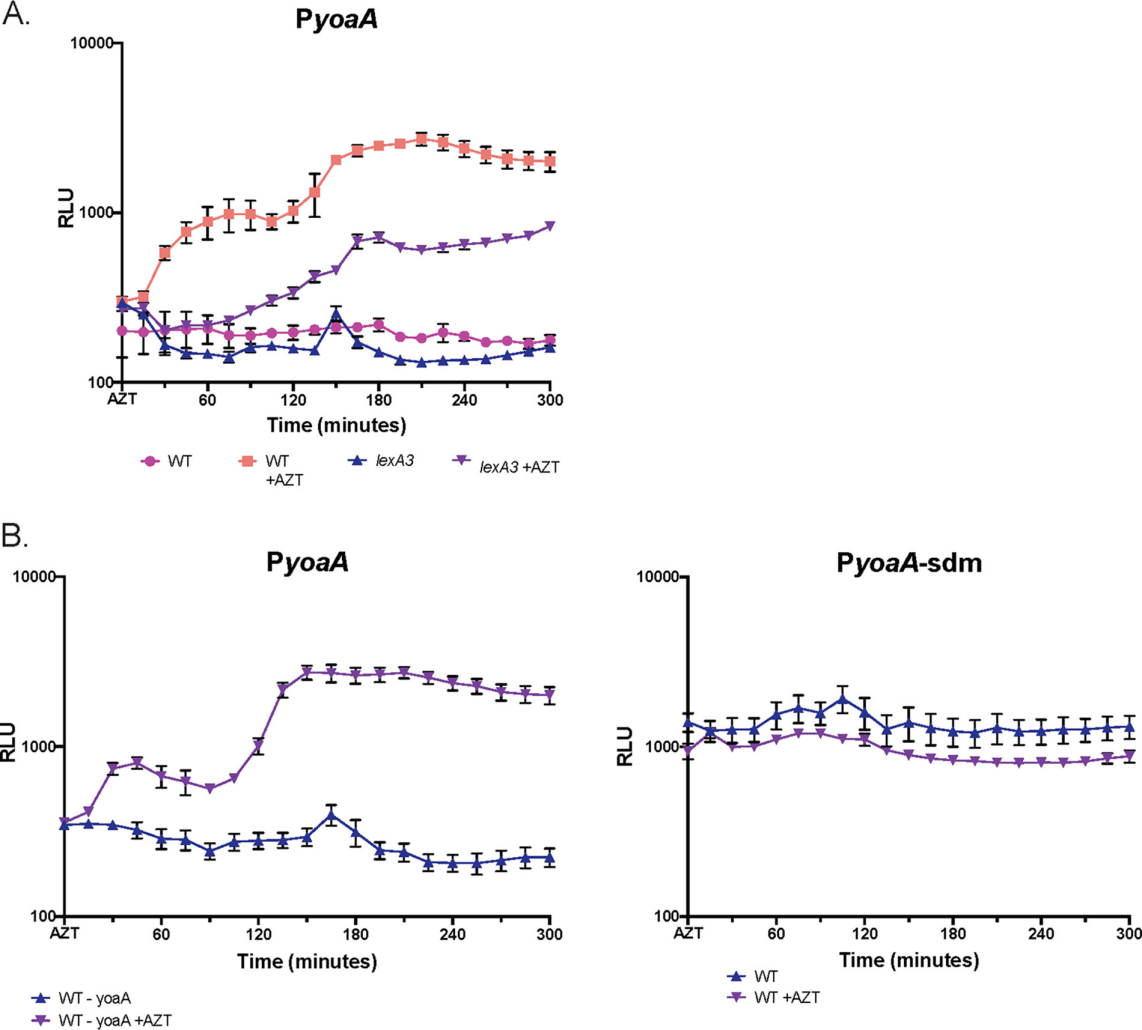
YoaA promoter (P*yoaA*) expression as measured by *lux* operon fusions. Cultures were grown in LB, with and without the addition of AZT at time zero. Values are expressed as relative luminescence units, with luminescence counts per minute divided by the OD_600_ of the culture at that time. The averages from 4 replicates are plotted, with error bars indicating standard errors of the means. (A) Expression from the *yoaA* 132-bp upstream intergenic region in wt strains, with and without AZT, compared to *lexA3* (noninducible), with and without AZT. (B, left) Expression of the *yoaA* upstream intergenic region in wt strains with and without AZT; (right) expression of the promoter region with a site-directed mutation, PyoaA-sdm, to remove the putative LexA box at position −24, with and without AZT, performed in parallel with the experiment on the left.

To study the regulation of the *yoaA* gene, we fused its upstream region (intergenic with divergently transcribed *yoaB*, with a length of 132 bp) to the Photorhabdus luminescens luciferase *luxCDABE* operon on a plasmid and measured the luminescence and optical density (OD) throughout the growth of the culture, with and without AZT addition (to 1.25 ng/ml) at time zero. This is a sublethal concentration of AZT for wt strains, where cells continue to proliferate. Expression was induced rapidly after the addition of AZT to the culture, with a peak approximately 60 min after treatment, as we have observed for other similar fusions to SOS-regulated promoter regions of *recA* and *dinB* (13). In contrast to those fusions, for *yoaA*::*lux*, we consistently saw a biphasic induction curve, with a slower second induction in late phases of growth, reaching a maximum approximately 150 to 180 min after treatment. A LexA mutation, *lexA3*, affecting the proteolytic cleavage site that renders the SOS response noninducible, negates most induction of expression by AZT, especially its expression soon after treatment, although the second slow phase of induction remained partially intact (Fig. 6A). At 180 min after AZT addition, expression in the *lexA3* genetic background is approximately 30% of that of the wt and 6-fold higher in treated than in untreated cells. Note that the data are normalized to the culture OD, so the slow increase in expression is not due to the expansion of the culture. In a second experiment, we compared the induction of *yoaA* expression by AZT from an intact upstream region with that of one in which we mutated the putative LexA box {replacing the two triplets of the invariant LexA box sequence CTG(N)_10_CAG with CCC(N)_10_GGG [(N)_10_ is TTCAAATCAA for *yoaA*]}. As expected for a LexA-repressed gene, we saw high constitutive expression in the absence of any damage, with no further increase by the addition of AZT (Fig. 6B).

### YoaA does not affect recombination.

**FIG 7:**
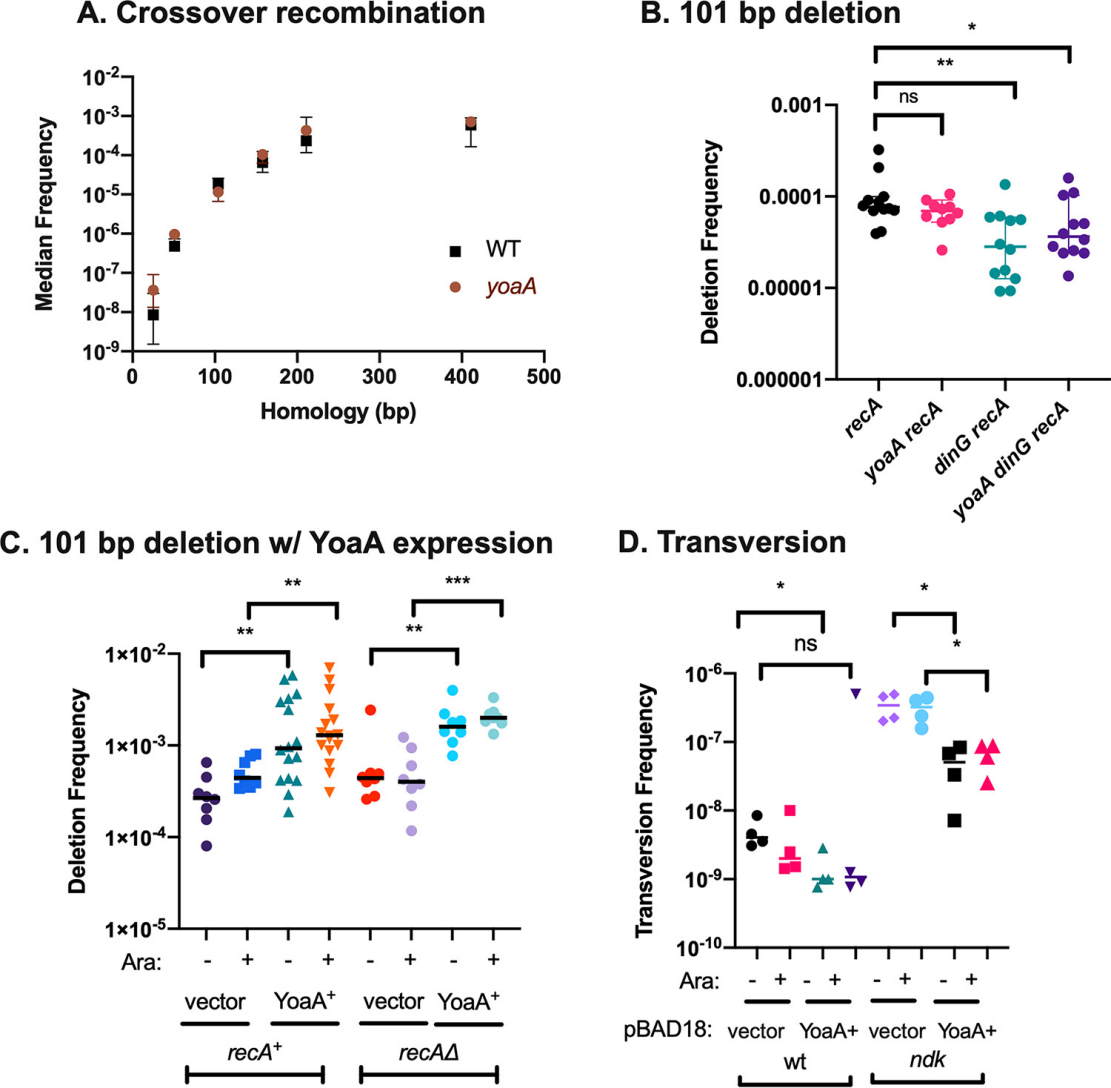
(A) Recombination frequencies of wt and *yoaA* strains with differing amounts of homology. (B) RecA-independent deletion frequencies between 101-bp tandem repeats in mutants of *dinG* and/or *yoaA*. Bars indicate median values, and the significance levels determined by a Mann-Whitney test are indicated with asterisks. The deletion assay plasmid is pSTL57. ns, not significant. (C) Deletion frequencies of wt or *recA*Δ strains carrying either the pBAD18 vector or a pBAD18 plasmid carrying *yoaA*^+^. Cultures were split and treated for 2 h with 0.2% arabinose (+) or not (−). The deletion assay plasmid is pSTL141. Bars indicate median values, and the significance levels determined by a Mann-Whitney test are indicated with asterisks. (D) A-to-T transversion frequencies in wt or *ndk* mutant strains carrying either the pBAD18 vector or *yoaA*, with and without arabinose induction.

Because a number of eukaryotic members of the XP-D/DinG helicase family affect homologous recombination or mutation rates, we examined whether the loss of *yoaA* had effects on several assays for recombination or genetic instability (Fig. 7). We measured crossover recombination using an assay previously developed that varies the amounts of homology between the recombining loci (25). Recombination at limiting homology often reveals genetic effects not apparent with larger homologies (25, 26). This assay primarily measures the RecFOR pathway of homologous recombination, which is believed to be the primary pathway in E. coli for recombination at single-strand gaps in DNA (reviewed in reference 27). We saw no influence of *yoaA* at any homology length using this crossover assay (Fig. 7A).

### YoaA and its paralog DinG promote template switching and genomic rearrangements.

Tandem direct repeats in DNA are unstable and prone to deletion. In E. coli, deletion between 101-bp tandem repeats occurs at a high frequency in the population during DNA replication, independent of the homologous-recombination factors, including RecA (reviewed in reference 28). Previous work supports a template-switching model for rearrangements between tandem repeats involving misalignment of the nascent strand.

**FIG 8:**
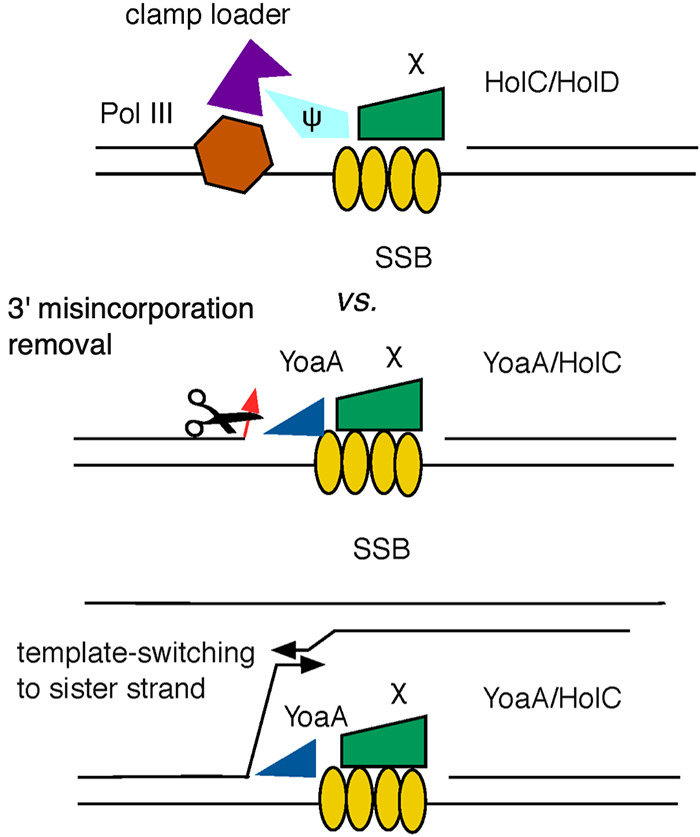
Diagram of HolC complexes and the reactions that they provoke. (Top) Through its interaction with SSB, HolC recruits HolD and the clamp loader and DNA Pol III replisome. (Middle) Alternatively, through its interaction with SSB, HolC recruits YoaA helicase, which unwinds the 3′ nascent strand, allowing exonucleases access for the removal of terminal misincorporation or AZT. (Bottom) recruitment and unwinding by YoaA through HolC may also initiate a template switch to the sister strand, producing repeat rearrangements and sister chromosome exchange.

Our model of how YoaA may facilitate replication fork repair (12) is that its 5′-to-3′ helicase activity unwinds the nascent 3′ terminus, allowing repair factors increased access to it (Fig. 8). In the template-switching model for deletion formation (reviewed in reference 28), a nascent 3′ terminus is unwound and mispairs with a second copy of the repeat, either on its downstream template or across the fork to the sister nascent strand (Fig. 8). It was therefore of interest to determine if YoaA or its paralog DinG had any effects on deletion frequencies between tandem repeats since they are candidates for the functions that could initiate a template switch. A knockout of *yoaA* did not affect RecA-independent deletion frequencies measured between 101-bp repeats in the *tetA* gene of plasmid pBR322 (Fig. 7B). However, we saw a modest but significant reduction of deletion frequencies of 2- to 3-fold in both the *dinG* single and *dinG yoaA* double mutants, suggesting that DinG promotes some spontaneous deletion events measured in this assay (Fig. 7B). The lack of an effect of *yoaA* does not necessarily mean that it does not stimulate deletion formation: its effects may be hidden by a larger contribution from DinG. To test whether YoaA could promote deletion when its expression was elevated, we cloned the gene under the control of a pBAD arabinose promoter. The expression of YoaA from this pBAD18 plasmid stimulated the deletion of 101-bp *tetA* repeats about 5-fold compared to control strains carrying the pBAD18 vector (Fig. 7C). We saw a substantial but perhaps smaller stimulation of deletion by *yoaA* even without induction with the *ara* promoter. We were unable to test the effects of DinG expression similarly due to its toxicity.

### YoaA may enhance proofreading.

If YoaA enhances access to the 3′ nascent strain, it may assist in the removal of incorrect nucleotides incorporated during replication, by either intrinsic (DnaQ-dependent) or extrinsic (DnaQ-independent) proofreading. Mutants in nucleotide diphosphate kinase, *ndk*, exhibit a mutator phenotype due to misincorporation caused by perturbations of deoxynucleoside triphosphate (dNTP) pools (29–31). A-to-T transversions, in particular, are elevated in *ndk* mutants; these are not affected by mismatch repair proficiency but are strongly influenced by proofreading by the DnaQ (ε) subunit of Pol III (30–32). Using specific *lacZ* revision assays, we examined the effects of YoaA expression on A-to-T transversions in the wt and *ndk* mutants. We observed a 120-fold stimulation of A-to-T transversion frequencies by the loss of *ndk* (Fig. 7D). Plasmid-expressed YoaA^+^ decreased transversion frequencies about 5- to 7-fold in *ndk* mutants. We saw a smaller reduction of transversion frequencies, 2- to 4-fold, by YoaA expression in wt strains. As we had seen previously for *yoaA* expression effects on deletion frequencies, induction of the pBAD promoter with arabinose had little effect.

## DISCUSSION

In this study, we examine further the genetic effects of the *yoaA* gene of E. coli. We mapped the residues of YoaA required for HolC binding to the C-terminal 18 amino acids of the protein, a region in which it is distinct from its paralog protein DinG. Mutation of two specific residues, R619 and T620, to alanine also abolished the interaction with HolC, as determined by yeast two-hybrid analysis. Mutants of *yoaA* are sensitive to the replication inhibitor AZT; the ability of YoaA to interact with the replisome protein HolC is likely required for AZT tolerance since YoaA R619A and T620A and C-terminal deletions fail to complement this defect and cause AZT sensitivity when these alleles are introduced into the natural *yoaA* gene on the chromosome. These mutations had only minor effects on YoaA protein accumulation *in vivo* and, based on the similarity of the protein to the DinG helicase for which there is structural information (23), likely affect a peripheral region of the helicase. Whether this interaction is merely a means for recruitment of the helicase to persistent ssDNA gaps, through a HolC/SSB interaction, or in some way alters the properties of the enzyme remains to be determined.

A knockout mutation of *yoaA* does not affect normal growth, whereas a knockout mutation of *holC* or *holD* produces slow growth and inviability, especially on rich media (24, 33). If YoaA/HolC forms an alternative complex to HolC/HolD, as we have proposed (16), increased expression of YoaA may lead to competition and a slow-growth phenotype by interfering with the formation of the growth-promoting HolC/HolD complex. The expression of YoaA from a strong promoter on a high-copy-number plasmid indeed inhibits growth, whereas the expression of C-terminally truncated YoaA does not. However, YoaA toxicity also requires the Walker A sequence involved in ATP binding and hydrolysis; this may indicate that elevated helicase activity, possibly unleashed from HolC, itself is deleterious. However, we do not know whether or how the YoaA Walker A box affects HolC binding or potential competition with HolD. In any case, the control of YoaA expression is likely critical to balancing repair versus replication functions.

We confirm that *yoaA* is a DNA-damage-inducible gene by promoter fusions to a luciferase (*luxCDABE*) operon, as suggested by previous microarray experiments, with and without UV exposure (7). We also demonstrate LexA regulation of the gene, with a noninducible *lexA3* allele reducing AZT induction and mutation of the predicted LexA box causing constitutively high expression. One advantage of *luxCDABE* luciferase fusions is that the expression signal can be detected in live cells (because the substrate is an endogenous metabolite), allowing us to monitor expression during the growth of the culture in the presence of a sublethal concentration of AZT. Following AZT treatment of an early-log-phase culture, *yoaA* shows an unusual bimodal induction of expression, with the first increase within 60 min, as is typical of SOS genes (see reference 13). AZT induces replication gaps and is a strong inducer of the SOS response via the RecAFOR pathway (13) that promotes the cleavage of the LexA repressor. In this previous study, AZT induction of *recA* promoter and *dinB* promoter fusions to luciferase was examined for a 2-h period after treatment. In addition to the immediate response, in this study, we observed a slower secondary increase as the culture began to approach stationary phase. Note that because AZT is incorporated during replication, it would not be expected to affect stationary-phase, nonreplicating cells. The *lexA3* mutation, which prevents its cleavage (34), abolishes the immediate mode of induction but does not negate the slow and more gradual induction at late phases of growth. We hypothesize that there is nutritional modulation of LexA repression of the *yoaA* gene such that induction of the gene is triggered more easily as the culture ages. We suspect that this is advantageous because the last rounds of replication may become more difficult to complete as cells begin to starve. Because this induction persists in the noncleavable *lexA3* strain and is not seen in the LexA box mutant, there may be an alternative mechanism to relieve LexA gene repression in addition to RecA-stimulated self-cleavage. To our knowledge, this phenomenon has not been reported previously. The generality and the mechanism of this second phase of induction remain to be determined. The regulation of YoaA is likely important because of its competition with HolD and the replisome for interaction with HolC/SSB, and YoaA expression may therefore interfere with replication.

Although YoaA promotes tolerance to ssDNA gaps caused by AZT incorporation into DNA, we do not find that it alters the efficiency of the RecAFOR pathway of homologous recombination during normal growth. YoaA had both positive and negative effects on genetic stability. Elevated expression of YoaA reduced A-to-T transversion mutations in a nucleotide diphosphate kinase *ndk* mutant, which is prone to misincorporation during replication due to perturbed dNTP pools (29–31). This observation is consistent with YoaA promoting proofreading, either intrinsic or extrinsic to Pol III, following nucleotide misincorporation. Conversely, elevated expression of YoaA promoted template switching that led to RecA-independent deletions formed between 101-bp tandem repeats. When we discovered that RecA-independent rearrangements were accompanied by crossing-over between sister chromosomes, we proposed that this pathway could function during replication gap repair and may be initiated by a DNA helicase (35). The data presented here suggest that both YoaA and DinG helicases are candidates for this role and that both can provoke template switching during replication. DinG appears to play a larger role during normal growth since its loss reduces constitutive levels of rearrangements, whereas YoaA loss does not but affects deletion rates when its expression is elevated. Both YoaA and DinG promote tolerance to AZT, but YoaA plays the larger role of the two. How these paralog proteins are specialized and their cellular roles remain to be explored more fully by further genetic and biochemical characterization.

## MATERIALS AND METHODS

### Strains, plasmids, and growth conditions.

For this study, we used Escherichia coli K-12 strain MG1655 as the wild type (wt) (*rph-1*) and isogenic strains STL3817 (*recA*::*cat*), STL9813 (*yoaA*Δ::FRT), STL9820 (*yoaA*Δ::FRT *recA*::*cat*), STL9824 (*dinG*::*kan recA*::*cat*), STL9822 (*yoaA*Δ::FRT *dinG*::*kan recA*::*cat*), STL22918 (*lexA3 malF3089*::Tn*10*), STL13722 (*lacZ*-T1385A *mphC281*::Tn*10*), STL15952 (*lacZ*-T1385A *mphC281*::Tn*10 ndk*Δ::FRT *kan*), STL23310 (*yoaA*-R619A::*cat*), STL23312 (*yoaA*-T620::*cat*), and STL23314 (*yoaA*Δ619–636::*cat*). LB (36), Lennox formulation, was used for standard growth medium. Plate medium included the addition of Bacto agar at 2%. For plasmid selection, the following antibiotics were employed at the indicated concentrations: ampicillin (Ap) at 100 μg/ml, kanamycin (Km) at 60 μg/ml, tetracycline (Tc) at 15 μg/ml, phleomycin (Phleo) at 5 μg/ml, and chloramphenicol (Cm) at 15 μg/ml. Strains were grown at 37°C. Minimal 0.2% lactose medium (37) was used to select Lac^+^ revertants in mutation assays.

For budding yeast, yeast extract-peptone-dextrose (YEPD) medium (complete) and dropout medium (synthetically deficient) were made according to previously reported recipes (38). Strains were incubated at 30°C.

Site-directed mutagenesis of plasmid pCA24N-YoaA+ was used to create YoaA mutants at specific residues. Plasmids and primers are listed in Tables 1 and 2. The forward primer was phosphorylated with T4 polynucleotide kinase (New England BioLabs) and used for high-fidelity PCR (see below) with its complement primer. Following DpnI digestion (New England BioLabs), the PCR product was purified (BioBasic Inc.), ligated with T4 DNA ligase (New England BioLabs), and transformed into the host strain XL1-Blue by electroporation (39). PCRs employed *Pfu* DNA polymerase from Agilent Technologies, using the guidelines provided by the manufacturer. To create each *yoaA* site-directed mutant, primers that were used are listed in Table 1. Plasmids (listed in Table 1) from bacterial transformants were isolated using BioBasic Inc. plasmid purification kits according to the manufacturer’s procedures. DNA sequence analysis (Genewiz) confirmed the presence of each particular *yoaA* site-directed mutant and no other change to the sequence.

**TABLE 1:** Plasmids^*a*^

Plasmid	pSTL plasmid designation	Reference, source, and/or description
pACYC184		41
pBAD18		45
pCA24N-holC+		42; ASKA 6×His collection clone, National Institute of Genetics
pCA24N-yoaA+		42; ASKA 6×His collection clone, National Institute of Genetics
pCL1		Plasmid control; Clontech, Takara Bio USA
pDEW201		43
pDONR201		Plasmid from Invitrogen, Thermo Fisher
pDONRZEO		Plasmid from Invitrogen, Thermo Fisher
pGADT7GW		Gateway Y2H plasmid from Addgene
pGADT7-T		Plasmid control from Clontech, Takara Bio USA
pGBKT7-53		Plasmid control from Clontech, Takara Bio USA
pGBKT7GW		Gateway Y2H plasmid from Addgene
pGBKT7-Lam		Plasmid control from Clontech, Takara Bio USA
pBR322 tetAdup101	57	44; deletion assay plasmid
pACYC184 tetAdup101	141	This study; deletion assay plasmid
pBR322d2	330	25; recombination plasmid 5′ Δ*tetA*
pACYC184up2	331	25; recombination plasmid *tetA*Δ3′ (25 bp of homology to pSTL330)
pACYC184up3	332	25; recombination plasmid *tetA*Δ3′ (51 bp of homology to pSTL330)
pACYC184up4	333	25; recombination plasmid *tetA*Δ3′ (104 bp of homology to pSTL330)
pACYC184up4.5	334	25; recombination plasmid *tetA*Δ3′ (158 bp of homology to pSTL330)
pACYC184up5	335	25; recombination plasmid *tetA*Δ3′ (211 bp of homology to pSTL330)
pACYC184up6	336	25; recombination plasmid *tetA*Δ3′ (411-bp homology to pSTL330)
pCA24N	393	Empty plasmid
pDONRZEO-holC+	404	16
pGADT7GW-holC+	409	16
pGBKT7GW-holC+	413	16
pDONRZEO-yoaA+	423	16
pGADT7GW-yoaA+	424	16
pGBKT7GW-yoaA+	425	16
pBAD18-YoaA	427	
pCA24N-yoaAΔ619-636	428	Created by whole-plasmid PCR primers 19 and 20
pCA24N-yoaA S613A	429	Created by whole-plasmid PCR primers 29 and 30
pCA24N-yoaA P615A	430	Created by whole-plasmid PCR primers 23 and 24
pCA24N-yoaA R619A	431	Created by whole-plasmid PCR primers 25 and 26
pCA24N-yoaA T620A	432	Created by whole-plasmid PCR primers 31 and 32
pCA24N-yoaA T620I	433	Created by whole-plasmid PCR primers 31 and 32
pCA24N-yoaA D622A	434	Created by whole-plasmid PCR primers 21 and 22
pCA24N-yoaA R625A	435	Created by whole-plasmid PCR primers 27 and 28
pCA24N-yoaA V627A	436	Created by whole-plasmid PCR primers 33 and 34
pCA24N-yoaA F629A	437	Created by whole-plasmid PCR primers 15 and 16
pGBKT7GW-yoaAΔ619-636	438	LR Gateway recombination, pSTL454 and pGBKT7GW
pGBKT7GW-yoaA R619A	439	LR Gateway recombination, pSTL452 and pGBKT7GW
pGBKT7GW-yoaA T620A	440	LR Gateway recombination, pSTL453 and pGBKT7GW
pGADT7GW-yoaAΔ619-636	441	LR Gateway recombination, pSTL454 and pGADT7GW
pET104.1 DEST yoaAΔ619-636	441	LR Gateway recombination, pSTL454 and pET104.1DEST
pGADT7GW-yoaA R619A	442	LR Gateway recombination, pSTL452 and pGADT7GW
pET104.1DEST-yoaA R619A	442	LR Gateway recombination, pSTL452 and pET104.1DEST
pGADT7GW-yoaA T620A	443	LR Gateway recombination, pSTL453 and pGADT7GW
pET104.1DEST yoaA T620A	443	LR Gateway recombination, pSTL453 and pET104.1DEST
pET104.1DEST-yoaA+	444	LR Gateway recombination, pSTL423 and pET104.1DEST
pDEW201-GW	447	pDEW201 with Gateway cassette B from Invitrogen-Thermo Fisher, inserted at the EcoK53i site
pDEW201-GW YoaAp	448	LR Gateway recombination, pSTL450 and pSTL447
pDEW201-GW YoaApSDM	449	Created by whole-plasmid PCR primers 37 and 38
pDONR201-GW YoaAp	450	BP Gateway recombination, pDONR201-GW and native *yoaA* PCR product from primers 35 and 36
pET104.1 (−ccdb)	451	Created by whole-plasmid PCR primers 39 and 40
pDONRZEO-yoaA R19A	452	BP Gateway recombination, pDONRZEO and *yoaA*-R619A PCR product
pDONRZEO-yoaA T20A	453	BP Gateway recombination, pDONRZEO and *yoaA*-T620A PCR product
pDONRZEO-yoaA Δ619-636	454	BP Gateway recombination, pDONRZEO and *yoaA*Δ619–636 PCR product
pCA24N-yoaA Δ632-636	456	
pCA24N-yoaA R628A	457	
pCA24N-yoaA K51R	458	Created by whole-plasmid PCR primer sets

a Y2H, yeast two-hybrid.

**TABLE 2:** Primers

Primer name	Primer no.	Sequence of primer
DB 09 YoaA b817R	1	CAGTCGCTGCCAAGACAGTTGTCG
DB 10 YoaA b693F	2	TACAAATCTTAAGCGATGTGATCC
DB 11 YoaA b1715F	3	GGTCGTTGTTTTATGCTTTGTACC
DB 12 YoaA b1825R	4	CTGACAAATTGCTGCAACAGTTGC
holC fusion attB1	5	GGGGACAAGTTTGTACAAAAAAGCAGGCTTCAAAAACGCGACGTTCTACCTT
holC fusion attB2	6	GGGGACCACTTTGTACAAGAAAGCTGGGTCTTATTTCCAGGTTGCCGTATT
holD KO confirm F	7	AGGTCATCCTGTAAGTCTCCGGCAAACAGA
holD KO confirm R	8	GATGTTCCAGCAGCGCCCTTCCCAATCCCT
pCA24N FOR seq	9	CATTAAAGAGGAGAAATTAACTATGAGAGG
pCA24NrrnBT1 REV s	10	ATGTGTCAGAGGTTTTCACCGTCATCAC
pGADT7_Seq_FOR	11	CGACTCACTATAGGGCGAGCG
pGADT7_Seq_REV	12	GTGCACGATGCACAGTTGAAGTGAAC
pGBKT7_Seq_FOR	13	GCCGCCATCATGGAGGAGCAG
pGBKT7_Seq_REV	14	CCCGGAATTAGCTTGGCTGCAAGC
YoaA F629A F	15	CGTGCGGTTCGTGCCCTTGCGATACCA
YoaA F629A R	16	TGGTATCGCAAGGGCACGAACCGCACG
yoaA fusion attB1	17	GGGGACAAGTTTGTACAAAAAAGCAGGCTTCACGGACGATTTTGCACCAGAC
yoaA fusion attB2	18	GGGGACCACTTTGTACAAGAAAGCTGGGTCTTACCTGGAGGATGGTATCGC
YoaA Truncation 3 R	19	TGGCGCGGGCGGCAGACTGGCGAG
YoaA Truncation F	20	TAACCTATGCGGCCGCTAAGGGTC
YoaAD622AF	21	CCACGCACCCGTGCCATTGCCCGTGCG
YoaAD622AR2	22	CGCGGGCGGCAGACTGGCGAGAAACGT
YoaAP615AF	23	CTCGCCAGTCTGGCGCCCGCGCCACGC
YoaAP615AR2	24	AAACGTCGCGCCGTAAGGACGCATCA
YoaAR619AFOR	25	CCGCCCGCGCCAGCCACCCGTGACATT
YoaAR619AREV	26	CAGACTGGCGAGAAACGTCGCGCCGTAAGGACGCATCACC
YoaAR625AF	27	CGTGACATTGCCGCTGCGGTTCGTTTC
YoaAR625AR2	28	GGTGCGTGGCGCGGGCGGCAGACT
YoaAS613AF	29	ACGTTTCTCGCCGCTCTGCCGCCCGCG
YoaAS613AR2	30	CGCGCCGTAAGGACGCATCACCA
YoaAT620AF	31	CCCGCGCCACGCGCCCGTGACATTGCC
YoaAT620AR2	32	CGGCAGACTGGCGAGAAACGTCGC
YoaAV627AF	33	ATTGCCCGTGCGGCTCGTTTCCTTGCG
YoaAV627AR2	34	GTCACGGGTGCGTGGCGCGGGCGGCA
yoaApromoterGWF	35	GGGGACAAGTTTGTACAAAAAAGCAGGCTTCCATTTTGTCCTCATTATACTTCCAT
yoaApromoterGWR	36	GGGGACCACTTTGTACAAGAAAGCTGGGTCACTACCCCCTGTTGATTTGAACAGG
yoaALexAp1	37	GCGCCCTCATCCTGACATAATGTCCCTTCAAATCAAGGGACGGTAGTGTGACGGAC
yoaALexAp2	38	GTCCGTCACACTACCGTCCCTTGATTTGAAGGGACATTATGTCAGGATGAGGGCGC
pET104.1 (-ccdb) Forward	39	ATGTCAGGCTCCGTTATACACAGCCAGTCT
pET104.1 (-ccdb) Reverse	40	TTCACCAGTCCCTGTTCTCGTCAGCAAAAG
YoaAK51RF	41	ACCGGTACGGGCAGAACCTACGCTTACCTG
YoaAK51RR	42	TCCTGCTTCCACCACCAGCGGCTGGCCTTTTTCT
YoaAR619AchromeSDM CAT	43	TTTCTCGCCAGTCTGCCGCCCGCGCCAGCCACCCGTGACATTGCCCGTGCGGTTCGTTTCCTTGCGATACCATCCTCCAGGTAAAAGCTTTTGATCGGCACGTAAGAGGTTCCAACTTT
YoaAT620AchromeSDM CAT	44	TTTCTCGCCAGTCTGCCGCCCGCGCCACGCGCCCGTGACATTGCCCGTGCGGTTCGTTTCCTTGCGATACCATCCTCCAGGTAAAAGCTTTTGATCGGCACGTAAGAGGTTCCAACTTTC
YoaAT3chromeCAT	45	TGATGCGTCCTTACGGCGCGACGTTTCTCGCCAGTCTGCCGCCCGCGCCATAAAAGCTTTTGATCGGCACGTAAGAGGTTCCAACTTTC
ReverseyoaAChromeSDMCAT	46	GGGTCTTCGTGCTTAGATCAATAAAAAGGCGCGCATCATACCATACTCCGTAACAAATTACGCCCCGCCCTGCCACTCATCGCAGTACTGTTGTAATTCATTAAGCATTCTGCC

The transfer of mutant alleles to the *yoaA* chromosomal locus was accomplished by lambda recombination using strain STL13149 (pSIM6 *mutS*::FRT *mhpC*::Tn*10*) (32) using a procedure described previously by Sawitzke et al. (40). PCR with primer 43, 44, or 45 and primer 46 (Table 2) was used to generate DNA fragments carrying the *cat* gene from plasmid pACYC184 (41), preceded by the appropriate mutant C-terminal region of *yoaA* and followed by homology to the gene downstream of *yoaA*, *tsaB*. The PCR fragment DNA was heat denatured before transformation into STL13149 by electroporation and with selection for Cm resistance. Transformants were grown at 42°C to cure pSIM6, and the alleles were moved into MG1655 by P1 transduction, producing strains STL23310 (*yoaA*-R619A::*cat*), STL23312 (*yoaA*-T620::*cat*), and STL23314 (*yoaA*Δ619–636::*cat*).

### Complementation and toxicity assays.

Plasmids, including the high-copy-number pCA2N vector, pCA2N-YoaA+ (42), and the indicated YoaA site-directed mutants, were introduced into strain STL9813 (*yoaA*Δ::FRT). Cultures were grown in LB containing Cm (LB +Cm) to log phase, at an OD at 600 nm (OD_600_) of 0.4, at which time they were serially diluted into 56/2 buffer and plated on LB medium containing 37.5 ng/ml AZT. For the toxicity assays, MG1655 strains carrying the pCA24N-derived plasmids were inoculated into LB −Ap medium in Costar 96-well assay plates and shaken at 37°C in a BioTek Cytation plate reader, measuring the OD_600_ every 15 min. When cultures reached an OD of 0.2 to 0.4, IPTG was added to 1/2 of the cultures to 1 mM, and the mixtures were incubated for an additional 3 h.

### Protein expression and Western blotting.

Wild-type biotin-binding domain (BBD)–YoaA (pET104.1DEST-yoaA), BBD-YoaA mutants (pET104.1 yoaA T620A, pET104.1 yoaA T3, and pET104.1DEST-yoaA R619A), and the empty pET104.1 vector (listed in Table 1) were expressed from the E. coli BL21(DE3) strain. The strains were grown in LB medium at 37°C with a final concentration of 100 μg/ml ampicillin, and the cells were induced with 1 mM IPTG for 2 h. The cells of all overexpressing strains were concentrated by centrifugation at 4,700 rpm for 30 min at room temperature, concentrated 1:100 in Tris-sucrose (50 mM Tris-HCl, 10% [wt/vol] sucrose [pH 7.5]), and stored at −80°C.

Crude cell extracts were prepared by lysozyme lysis using 1 μM dithiothreitol (DTT) and 0.1 mg/ml lysozyme (United States Biochemical) in Tris-sucrose. After a 5-min incubation on ice, NaCl was added to a final concentration of 0.2 M, and the extract was incubated for an additional 25 min, after which the cells were heat shocked at 37°C for 15 s and then transferred to ice for 30 s. Following two heat shocks, the lysed cells were centrifuged, and the crude lysate supernatant was collected. BBD-YoaA protein samples were combined with an equal volume of 2× FSB (0.12 M Tris-HCl [pH 6.8], 3.8% SDS, 19% glycerol, 1.43 M β-mercaptoethanol, 1 mg/ml bromophenol blue). Samples were subjected to polyacrylamide gel electrophoresis in 12% polyacrylamide gels and transferred to a polyvinylidene difluoride (PVDF) membrane utilizing a Bio-Rad transblot apparatus at 100 V and 400 mA for 75 min. Western blot analysis was performed according to the QIAexpress detection kit protocol (Qiagen), with the following modifications: BBD-YoaA was detected with a 1:1,000 dilution of neutravidin antibody (Thermo Fisher) in 10% nonfat milk for 1 h. The gel was then washed four times for 10 min with Tris-buffered saline–Tween 20 (TBS-T) wash buffer (20 mM 1 M Tris-HCl [pH 7.5], 500 mM NaCl, 0.05% Tween 20, 0.2% Triton X-100). Imaging was performed on a Bio-Rad ChemiDoc system.

### Construction of GAL4 activation domain and binding domain fusions to HolC and YoaA for the yeast two-hybrid system.

Bacterial colony PCR with Phusion high-fidelity DNA polymerase (New England BioLabs) was used to obtain the wild-type alleles of *holC* and *yoaA* for the construction of GAL4 fusions for the yeast two-hybrid analysis as previously described (16). Gateway cloning technology from Invitrogen was used to transfer the mutations created in pCA24N-YoaA to the GAL4 activation and binding domain plasmids pGADT7GW and pGBKT7GW, respectively. Primers were used for high-fidelity PCR using *Pfu* DNA polymerase obtained from Agilent. Subsequent PCR mixtures were subjected to DpnI digestion to destroy the template plasmid. Following purification of the PCR products (BioBasic kits), *yoaA* mutant fragments (*attB1-yoaA* mutant-*attB2*) were cloned into pDONRZEO using the BP Clonase II enzyme and subsequently cloned into either the pGADT7GW or pGBKT7GW vectors using LR Clonase II.

### Yeast two-hybrid analysis.

We used the procedures and controls from the Matchmaker gold yeast two-hybrid system from TaKaRa Bio, with the following modifications. Negative controls consisted of the ones suggested for the Matchmaker gold yeast two-hybrid system with the addition of *holC* and *yoaA* activation domain hybrid plasmids paired with the binding domain empty plasmid. Similarly, *holC* and *yoaA* binding domain plasmids were paired with the activation domain empty plasmid vector. Single colonies of all controls and combinations of the activation and binding domain plasmids were grown in 5 ml of either leucine or leucine and tryptophan dropout medium for 20 h. Following such incubation, cultures were diluted 1:5 and 1:50 in sterile water. A total of 1/20 of this dilution was plated onto the following media: YEPD medium lacking leucine and tryptophan, YEPD medium lacking leucine and containing tryptophan, and YEPD medium lacking histidine and adenine. YEPD medium is a universal medium in which all cultures will grow. Plates that lacked either leucine, tryptophan, or both leucine and tryptophan were used as the controls to test plasmid retention. Histidine-deficient plates were used to test for lower-stringency protein-protein interactions, while adenine-deficient plates were used to test for a higher stringency. Plates were incubated for 2 or 3 days at 30°C.

### Luciferase gene expression assays.

Luciferase fusion construct plasmids were based on plasmid pDEW201 (43) with an inserted Gateway cloning *attR* site-specific recombination cassette. Plasmid construction was completed using Gateway cloning (Life Technologies) from PCR-amplified products with the primer pair yoaApromoterGWF (5′-GGGGACAAGTTTGTACAAAAAAGCAGGCTTCCATTTTGTCCTCATTATACTTCCAT-3′) and yoaApromoterGWR (5′-GGGGACCACTTTGTACAAGAAAGCTGGGTCACTACCCCCTGTTGATTTGAACAGG-3′). Products were recombined with the BP reaction mixture into the pDONR201 Gateway plasmid vector. Verified pDONR201GW-yoaAp constructs were recombined using the LR reaction into the Gateway pDEW201 Ap LuxCDABE vector. To create yoaApSDM with the mutated LexA box, site-directed mutagenesis (QuikChange; Agilent Technologies) of pDEW201GW-yoaAP was conducted according to the manufacturer’s instructions, using overlapping PCR primers with the target mutation, yoaALexAp1 (5′-GCGCCCTCATCCTGACATAATGTCCCTTCAAATCAAGGGACGGTAGTGTGACGGAC-3′) and yoaALexAp2 (5′-GTCCGTCACACTACCGTCCCTTGATTTGAAGGGACATTATGTCAGGATGAGGGCGC-3′), and amplification with the Phusion high-fidelity DNA polymerase PCR kit (New England BioLabs). Constructs were sequence verified. The pDEW201 vector or pDEW201 carrying the *yoaA* promoter region (pDEW201-GW YoaAp) or mutated at the LexA box (pDEW201-GW YoaApSDM) was introduced into strain MG1655 (wt) or STL22918 (*lexA3 malF3089*::Tn*10*) by electroporation, selecting for Ap resistance.

The luminescence and OD_600_ were measured using a BioTek Cytation 1 plate reader and a Costar 96-well assay plate (treated polystyrene, black plate, and clear bottom). Colonies were inoculated in LB medium in tubes, with shaking, until the OD_600_ reached 0.5, after which they were diluted 1:100 in LB and grown again to ensure log-phase growth. In the 96-well plates, cells were diluted 1:100 and grown for 2 h before being treated with 1.25 ng/ml AZT. Bioluminescence was measured and normalized to the OD_600_, yielding relative luminescence units (RLU) every 15 min, and data are averages of results from 4 independent replicate cultures.

### Recombination, mutagenesis, and deletion assays.

Recombination assays were performed as previously described (25), using pairs of compatible plasmids carrying 5′-deleted *tetA* or 3′-deleted *tetA*, with homologies between the two ranging from 25 to 411 bp. Crossing-over between these regions of homology generated an intact *tetA* gene and is selected by the acquisition of Tc resistance. Pairs of plasmids were introduced into MG1655 and *yoaA* (STL9813) mutant strains by electroporation, selecting for Ap and Cm resistance, and assayed in parallel for the frequency of Tc resistance in the populations on multiple days. Transversion mutagenesis assays were performed using previously described alleles of chromosomal *lacZ* that revert to Lac^+^ only by an A-to-T transversion (32) in the wt (STL13722) or *ndk* (STL15952) background. The vector plasmid (pBAD18) or a plasmid expressing *yoaA* (pBAD18-YoaA) was introduced into these backgrounds. Cultures were grown in LB Ap and then split, with expression induced in one by the addition of 0.2% arabinose. Cultures were grown for an additional 2 h and then diluted and plated on minimal lactose medium to count Lac^+^ revertants and LB to count the total number of cells in the culture. Plasmids pSTL57 (Ap^r^; ColE1 replicon [44]) and pSTL141 (Cm^r^; p15a replicon) were used to measure deletion formation between 101-bp tandem repeats in *tetA* as previously described (44). For experiments examining the effects of *dinG* and/or *yoaA* knockout mutations, the pSTL57 plasmid was introduced into strains STL3817 (*recA*::*cat*), STL9813 (*yoaA*Δ::FRT), STL9820 (*yoaA*Δ::FRT *recA*::*cat*), STL9824 (*dinG*::*kan recA*::*cat*), and STL9822 (*yoaA*Δ::FRT *dinG*::*kan recA*::*cat*). Cultures in LB Ap were grown to an OD of 0.4, serially diluted, and plated on LB Ap and LB Ap Tc. For experiments with induced expression of YoaA, pBAD18-YoaA or pBAD18 (Ap^r^; ColE1 [45]) was introduced into the MG1655 (wt), STL9813 (*yoaA*Δ::FRT), STL3817 (*recA*::*cat*), and STL9820 (*yoaA*Δ::FRT *recA*::*cat*) strains carrying the pSTL141 deletion reporter. Cultures grown in LB Ap Cm were split, with expression induced in one by the addition of 0.2% arabinose. Cultures were grown for an additional 2 h, diluted, and plated on LB Ap Cm Tc.
