# Supplementary material for: Genetic Analysis of DinG Family Helicase YoaA and Its Interaction with Replication Clamp Loader Protein HolC in Escherichia coli
Source: J Bacteriol. 2021 Aug 20;203(18):e00228-21. doi: 10.1128/JB.00228-21 (PMC8378479; doi:10.1128/JB.00228-21)
Supplement: Supplemental file 1 — Fig. S1. Download JB.00228-21-s0001.pdf, PDF file, 0.03 MB [file jb.00228-21-s0001.pdf]

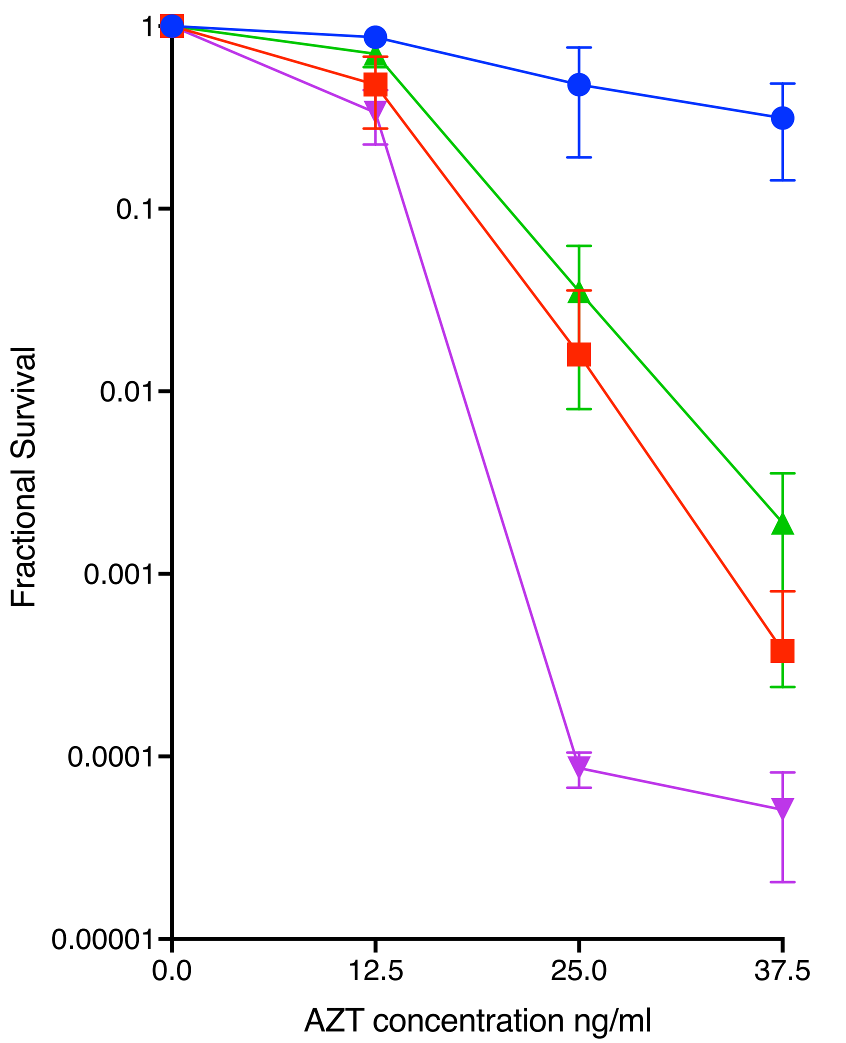

Supplemental Figure 1. AZT survival of wt (MG1655, blue circles), or *yoaA*Δ619-636 (STL23314, purple triangles), *yoaA* R619A (STL23310, red squares) *yoaA* T620A (STL23312, green triangles) mutants at the natural *yoaA* chromosomal locus.
